# Analysis of the Adequacy of Prehospital Emergency Medical Services Use of Patients Who Visited Emergency Departments in Korea from 2016 to 2018: Data from the National Emergency Department Information System

**DOI:** 10.1155/2021/6647149

**Published:** 2021-04-16

**Authors:** Sung Joon Park, Jung-Youn Kim, Young-Hoon Yoon, Eu Sun Lee, Hyun-Jin Kim, Seoung Bum Kim, Hyun Gu Kahng

**Affiliations:** ^1^Department of Emergency Medicine, Korea University College of Medicine, Seoul, Republic of Korea; ^2^School of Industrial Management Engineering, Korea University, Seoul, Republic of Korea

## Abstract

**Introduction:**

Proper ambulance use is important not only due to the patient's transport quality but also because of the need for efficient use of limited resources allotted by the system. Therefore, this study was conducted to check for overuse or underuse of the ambulance system by patients who visited the emergency department (ED).

**Methods:**

In this study, a secondary data analysis was conducted using the existing database of the National Emergency Department Information System with all patients who visited EDs over the three-year study period from 2016 to 2018. The study subjects were classified into the following groups: (1) appropriate Emergency Medical Services (EMS) usage; (2) appropriate no EMS usage; (3) underuse; and (4) overuse groups.

**Results:**

Of 18,298,535 patients, 11,668,581 (63.77%) were classified under the appropriate usage group, while 6,629,954 (36.23%) were classified under the inappropriate usage group. In the appropriate EMS usage group, there were 2,408,845 (13.16%) patients. In the appropriate no EMS usage group, there were 9,259,706 (50.60%) patients. As for the inappropriate usage group, there were 5,147,352 (28.13%) patients categorized under the underuse group. On the other hand, there were 1,482,602 (8.10%) patients under the overuse group.

**Conclusion:**

There are many patients who use ambulances appropriately, but there are still many overuse and underuse. Guidelines on ambulance use are necessary for the efficient use of emergency medical resources and for the safety of patients.

## 1. Introduction

Patients are brought to emergency departments (EDs) through a variety of modes of transportation. The Emergency Medical Service (EMS) system of Korea started in 1982. The system uses ambulances to transport severely critical or injured patients to the hospital [1, 2]. However, patients sometimes opt to come to EDs by foot or in their own cars depending on their situation or available mode of transportation. The patients arriving at EDs are classified according to their severity, which is used for the prioritization of treatment.

For patients with a high severity level, it is desirable to use an ambulance that can safely transport the patient and support appropriate emergency treatments. Therefore, it is reasonable for patients with a high severity level to use the EMS system in situations where it is necessary. If transportation of a severe patient without any appropriate treatment takes too long, the patient's condition will likely worsen. The EMS system is a medical resource which operates with limited manpower and funds. For this reason, if all patients are brought to EDs using the system, this results in an unnecessary waste of resources and patients will be unable to receive effective treatment. Therefore, it is desirable for patients with mild illnesses to use their own vehicles to arrive at the hospital [3–7].

It is important for patients coming to EDs to use the appropriate mode of transportation, taking into consideration their treatment and the efficient distribution of medical resources. Although many patients visit EDs through various modes of transport, there has been no evaluation as to whether these transport modes have been used appropriately. Therefore, the researchers tried to analyze the appropriateness of modes of transport to the hospital using data from patients who had visited EDs in Korea over a period of three years.

## 2. Materials and Methods

### 2.1. Study Design and Database

In this study, a secondary data analysis was conducted using the existing database of the National Emergency Department Information System (NEDIS). NEDIS, an emergency department information network operated by the government (the Ministry of Health and Welfare), is managed by the National Emergency Medical Center. Since the execution of the system in 2003, it has collected clinical and administrative data of all the patients who visited EDs across the nation. Korea has also implemented national medical insurance which covers 98% of the Korean population. Therefore, the data collected in Korea is substantial. Emergency medical centers in the country undergo evaluation once a year to be approved as an official organization, and, on principle, automatically transmit all digitalized data for the items requested by NEDIS. Therefore, the data utilized in this study are considered to have incorporated all the data of EDs in Korea [8–10]. The Korean government has 36 regional emergency medical centers, 117 local emergency medical centers, and 258 local emergency medical institutions designated for use.

### 2.2. Data Collection

Based on three years of data collected from January 1, 2016, to December 31, 2018, a retrospective data analysis had been conducted with all patients who visited EDs. After the application for data opening, the patients' information including their age, gender, Korean Triage and Acuity Scale (KTAS) score, result of ED (discharge or admission), mechanism of injury, insurance type, disease or injury, intent of injury, route of arrival, mode of transport, mental status at arrival, type of emergency medical center, arrival time, arrival day, and arrival month was extracted and analyzed. Patients whose medical records were lacking were excluded since it was impossible to find answers to these items elsewhere. Data about all the ED patients (including young children and adults) in the National Emergency Department Information Network were collected.

### 2.3. Variables

The Korean Triage and Acuity Scale (KTAS), which has been used by national EDs for triage classification since 2016, was applied in this study. According to KTAS, ED patients are classified into five levels. The lower the level is, the more urgent the condition of a patient. A patient in level 1 requires emergent treatment and is in a life- or limb-threatening condition. Typical patients in level 1 have symptoms such as cardiac arrest or apnea. A patient in level 2 faces a potential threat against their life, limbs, and body functions and therefore requires immediate treatment. The patients in the case have suspicious symptoms such as myocardial infarction and cerebral stroke. For patients in level 3, it is necessary to take into account the potential possibility of their condition developing into one that requires treatment. A patient in level 3 needs to be treated or reexamined within 1-2 hours, taking into consideration their age, level of pain, and the possibility of deterioration/complications. A patient in level 5 is not in an urgent situation. Such patients may have a chronic disorder and have a low possibility of deterioration [11–14].

One primary outcome of the study was the verification of the comparison of the appropriateness of patients' transport modes based on their KTAS levels. Transport was deemed “appropriate” if patients with KTAS levels of 1 to 3, urgent patients, used the EMS system that is defined as a transport using ambulance whether public, private, or hospital, while patients with KTAS levels of 4 to 5, less urgent patients, did not use EMS system. “Inappropriate use” included patients who fell into the overuse or underuse group. Patients were classified under the “underuse group” if they had a KTAS level of 1 to 3 but did not use the EMS system, while patients with KTAS levels of 4 to 5 who used the EMS system were classified under the “overuse group.” Basic characteristics of the patients including their age, gender, result of ED (discharge or admission), mechanism of injury, insurance type, disease or injury, intent of injury, route of arrival, mode of transport, mental status at arrival, type of emergency medical center, final triage level, arrival time, arrival day, and arrival month were analyzed.

### 2.4. Statistical Analysis

Data were stored using SPSS (version 20.0; IBM SPSS, Armonk, NY, USA) and imported into Excel (Microsoft Corporation, Redmond, WA, USA). All statistical analyses were performed using Python (version 3.6; Python Software Foundation, Wilmington, DE, USA) with the Pandas package (version 1.0.0; NumFOCUS, Austin, TX, USA). Unless otherwise specified, statistical significance was indicated by a *p* value of <0.05.

### 2.5. Ethics Statement

This research received approval from the institutional review board of the Korea University Guro Hospital (no. 2020GR0010). The requirement of informed consent from the participants was waived by the board.

## 3. Results

### 3.1. General Characteristics of ED Patients

**Figure 1:**
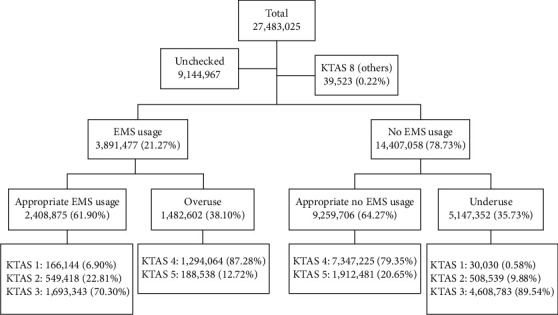
Flow chart describing case enrollment. Of 18,298,535 patients, 11,668,581 patients (63.77%) had appropriate usage and 6,629,954 (36.23%) had inappropriate usage. Around 2,408,845 (13.16%) belonged to the appropriate (ambulance used) group, 5,147,352 (28.13%) belonged to the underuse group, 9,259,706 (50.60%) belonged to the appropriate (no ambulance used) group, and 1,482,602 (8.10%) belonged to the overuse group.  ^*∗*^KTAS: Korean Triage and Acuity Scale.

During the research period from 2016 to 2018, a total of 27,483,025 patients had visited EDs across the nation (Figure 1 and Table 1). Of these patients, 9,144,967 had no KTAS levels indicated. Cases without KTAS levels were found in local institutions which had no obligation to enter KTAS information. The KTAS input rate according to the type of emergency medical center is as follows: local institutions had an input rate of 14.00%, local centers had a rate of 98.97%, and regional centers had a rate of 99.98%. The total number of cases where KTAS levels were indicated was 18,338,058, among which 196,144 were found to be patients with KTAS level 1, accounting for 1.07% of the population. There were 1,057,957 (5.77%) patients with KTAS level 2, 6,302,126 (34.37%) patients with KTAS level 3, 8,641,289 (47.12%) patients with KTAS level 4, and 2,101,019 (11.46%) patients with KTAS level 5. There were 39,523 (0.22%) patients with KTAS level 8, which codes for cases accepted for other reasons. All in all, this study was conducted using 18,298,535 cases, excluding cases without KTAS levels and those with KTAS level 8 (others). The average age of the patients was 39.99 years with a standard deviation of 24.21 years. 51.49% of the ED patients were male.

### 3.2. Appropriateness of EMS System Use of ED Patients and General Characteristics of Each Group

The ED patients were largely divided into the appropriate usage group and inappropriate usage group and then subsequently categorized into a total of four groups (Table 2).

Of 18,298,535 patients, 11,668,581 (63.77%) were classified under the appropriate usage group, while 6,629,954 (36.23%) were classified under the inappropriate usage group. The number of patients under the appropriate usage group was larger than that of the inappropriate usage group.

In the appropriate usage group, there were 2,408,875 (13.16%) patients with KTAS levels 1 to 3 who used the EMS system, their average age was 58.20 years old (SD = 22.87), and 53.81% were men. In the appropriate usage group, there were 9,259,706 (50.60%) patients with KTAS levels 4 to 5 who did not use the EMS system. Their average age was 34.15 years old (SD = 23.90), and 51.28% were male. As for the inappropriate usage group, there were 5,147,352 (28.13%) patients categorized under the underuse group (KTAS levels 1 to 3 who did not use the EMS system). Their average age was 39.60 years old (SD = 26.89) and 49.83% were male. On the other hand, there were 1,482,602 (8.10%) patients under the overuse group of patients (KTAS levels 4 to 5 who used the EMS system). Their average was 50.46 years old (SD = 23.36) and 53.89% were male.

### 3.3. Characteristics of Patients in Appropriate EMS Usage Group

**Figure 2:**
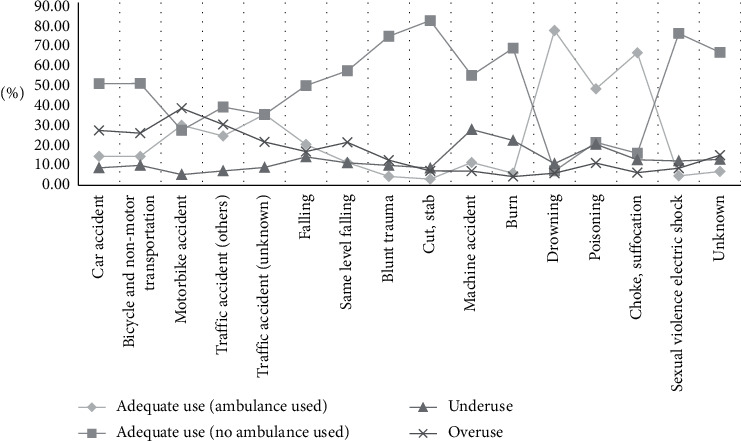
Comparison by the mechanism of injury. Regarding the mechanism of injury, there are higher rates of overuse in cases due to the traffic accident-related group.

**Figure 3:**
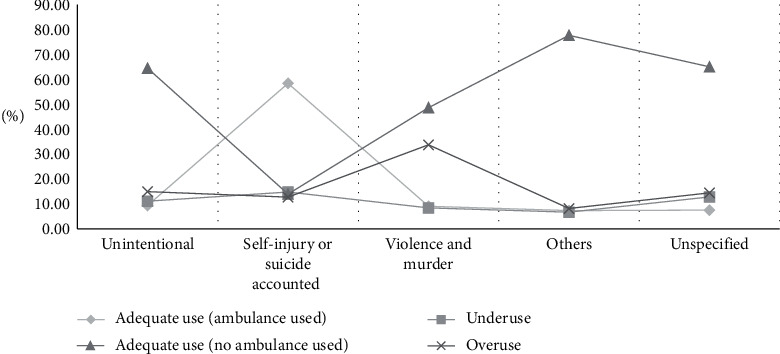
Comparison by the intention of injury. In the group of violence, overuse is more and there was a higher rate of appropriate use due to self-injury.

**Figure 4:**
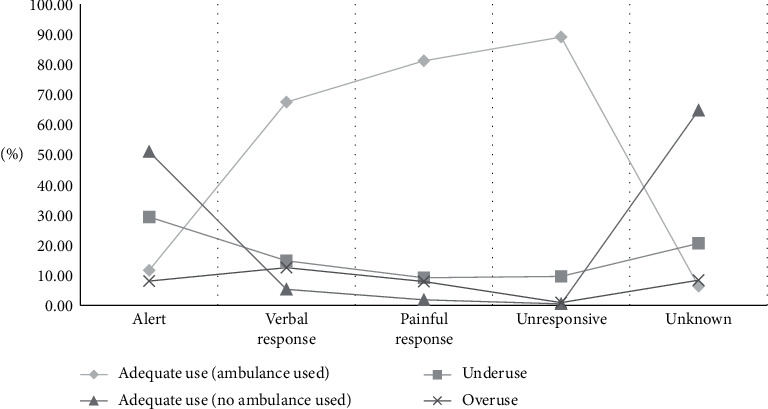
Comparison by initial mental status. By initial mental status, the pattern of ambulance use is different. In the alert group, there was a higher rate of underuse.

**Figure 5:**
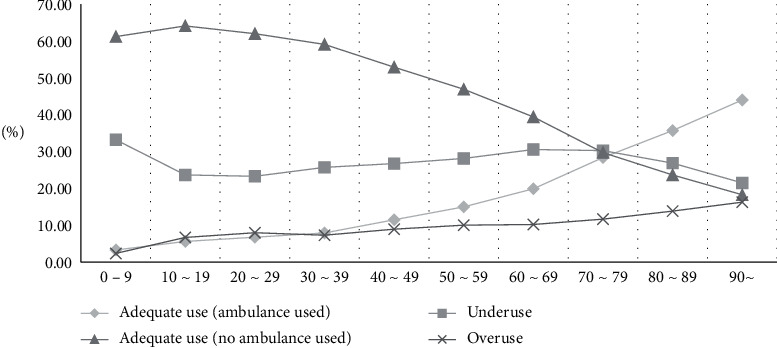
Comparison by age group. Older people use the ambulance more than the young. Under 9, underuse is the highest.

With regard to the final disposition result of ED patients, the cases of death at EDs accounted for the highest rate, or 87.41% (Table 2). Regarding the mechanism of injury, there was a high rate of cases resulting from poisoning, suffocation, or choking (Figure 2). For the intent of injury, cases of self-injury or suicide accounted for 58.40% of the cases, while other cases had relatively similar rates with one another (Figure 3). For the response status at arrival, several patients who used the EMS system were either capable of verbal response or responding to pain, or were unresponsive, which classified their cases as urgent. Regarding types of insurance, patients with medical care type 1 insurance (27.5%) and industrial injury insurance (21.81%) were more likely to use ambulances appropriately. The appropriate usage rate of 119 ambulances was lower than that of other ambulances and of ambulances belonging to medical centers. Appropriate usage rates were also low in cases where the patients' mental status was alert (Figure 4), when patients visited emergency medical institutions, and when patients were brought to the ED between 20:00 and 22:00, on Sundays, or in December. Lastly, the older the patients, the higher the rate of ambulance use (Figure 5).

### 3.4. Characteristics of Patients in Appropriate No EMS Usage Group

Of the group of patients who had appropriate responses to emergent situations, 9,259,706 did not use the EMS system, and among them, 3,183,786 had no diseases (Table 2). The group comprised 51.01% ED patients whose mental status was alert (Figure 4). In addition, 7,347,225 patients in this group had KTAS level 4 (less urgent). Regarding the ED visit time, the rate of visits of patients to the ED in the morning or during daytime in this group was relatively low; however, this gradually rose in the evening (Table 3).

### 3.5. Characteristics of Underuse Patients

There were 5,147,352 in the underuse group (Table 2). With regard to ED disposition, 11.2% of patients who died failed to use the EMS system appropriately. As for the mechanism of injury, the underuse rate was the highest in cases of mechanical accidents, burns, and poisoning, in decreasing order (Figure 2). Regarding the type of insurance, the underuse rate was higher in cases of patients with private insurance compared to cases of patients with other types of insurance. Regarding disease or injury, the underuse rate was higher in cases of disease compared to that in cases of trauma. With regard to the intent of injury, the underuse rate was low in cases of violence and murder, whereas the underuse rate was high in cases of self-injury and suicide (Figure 3). The underuse rate of ED patients with an alert mental status was 29.33% (Figure 4). The underuse rate was the highest in the group of patients with KTAS level 3. Furthermore, the underuse rates were high when patients were brought to the ED in the morning from 10:00 to 12:00 and did not differ depending on the days of the week and months (Table 3). The younger the patient, the higher the rate of underuse, especially in patients under 10 years old (Figure 5).

### 3.6. Characteristics of Overuse Patients

There were 1,482,602 patients in the overuse group (Table 2). The overuse rate was 37.96% in patients who were brought to the ED due to motorcycle-induced accidents. Mostly, traffic accident victims used ambulances. In the same context, the overuse rate was higher in cases of patients who had automobile insurance (28.20%) as compared to those with other types of insurance. The overuse rate was higher in cases of trauma as compared to cases of diseases. Regarding intent of injury, the overuse rate was higher in cases of violence and murder (33.77%) as compared to other cases (Figure 3). The overuse rate was the highest when the patients were brought to the ED at dawn from 1:00 to 4:00 AM. On the other hand, the rates were lower on Sundays and did not largely differ depending on the month (Table 3). The older the patients, the higher the overuse rate (Figure 5).

## 4. Discussion

In this study, a complete enumeration survey was conducted with patients who had visited EDs for three years based on the systemized data registry of registered EDs in the country. The patterns of transport modes that ED patients used to arrive at the hospital were analyzed. Therefore, this study revealed the general characteristics of ED patients and their use of ambulances to arrive at the hospital.

Generally, those who overused the EMS system were males, old patients, traffic accident victims, those who had accident-related insurance, and/or trauma victims. Patients with lower rates of EMS system use included females, those who were brought to the ED due to disease, those with an alert mental status, and KTAS level 3 patients. The EMS system also had lower rates of use in the evening and higher rates of use at dawn. The overuse rate was low on Saturdays and Sundays, while the rates of appropriate usage were higher on Saturdays and Sundays than on other days. The results also showed that there are higher rates of EMS system usage in cases due to external factors such as trauma or accidents. Given that there are more male patients in the ED and there are higher rates of visits on weekdays, it is fair to say that rates differ depending on the patients' gender and the day of the week [15, 16].

Korea is a country with a well-developed public ambulance system established by the government. In Korea, the EMS system is offered as a free public transportation mode to the hospital. Since there are no clear criteria and set limitations of 119 ambulance use, the usage rate differs depending on the patients' selection. This study revealed the characteristics of patients who used the EMS system and found that patient characteristics may influence their rate of use of the EMS system. Therefore, it is very important to consider the patients' characteristics in the efficient operation of a medical system.

The EMS system is operated using limited resources. For this reason, the EMS system is already quantitatively controlled and efficiently managed, and efforts should focus on improving its quality. Moreover, it is necessary to evaluate the Korean EMS system to find if it is appropriately used, to evaluate it annually, and to improve people's perceptions of EMS system use. Therefore, it is necessary to analyze the patient's personal characteristics (e.g., underlying diseases, ability to move) and to conduct an in-depth analysis on the reasons for their use or disuse of the EMS system [7].

Since there are several factors to consider such as socioeconomic, cultural factors, and patients' personal situations, it is very difficult to analyze the adequacy of EMS system use and establish unified criteria. The use of the EMS system largely depends on a patient's subjective judgment [17–20]. For this reason, it is hard to set clear criteria for using the system. Morris and Cross [21] classified the use of ambulances into the following types: justified, possibly warranted, and unnecessary. Evaluation criteria were analyzed in consideration of the patients' diagnosis, age, and socioeconomic status. Ambulance use by elderly patients or patients with mild head injuries was classified as justified, while use by those with head lacerations was classified as unnecessary. The time slots available for treatments as outpatients, ankle sprain, nausea, and nonbleeding injuries were classified as unnecessary. Of 1,000 study subjects, the ambulance use of 381 patients was classified as justified, 102 as possibly warranted, and 517 as unnecessary.

In a report by Brown and Sindelar [22], transport appropriateness was judged in consideration of a patient's main symptom, whether or not there was a need for resuscitation in the ambulance, type of medical insurance, severity of injury, and capability of movement, diagnosis, and whether or not the patient needed to be hospitalized. If a patient's main symptom is not urgent and if the patient can move and does not require hospitalization or resuscitation, the patient is judged to need no ambulance. The appropriate usage rate differed depending on the type of insurance. The appropriate usage rate in cases of patients with private medical insurance was 78.8%. On the other hand, the inappropriate usage rate in cases of patients who had no copayment was 85.3%. The usage rate also differed depending on the copayment state. In Korea, it could be assumed that the rate of overuse is high since patients have no copayment. However, the overuse rate was only 8.10%, which was lower than the underuse rate (28.13%). This showed that other factors such as the determination of whether to use the EMS system is correct or not, the idea that arriving at the hospital will be faster than calling EMS and considering not only arriving at the hospital but also returning home, as well as costs, affect the use of EMS system.

Mooney et al. [5] reported that even if the cost is free, the use of ambulance services may not be positively accepted by the public. According to this research in Ireland, 40.1% of acute coronary syndrome (ACS) patients used ambulances. This is similar to the result of this study wherein patients who are alert and have internal diseases have lower rates of ambulance use. However, it is better to use ambulances for patients with ACS since these cases are considered urgent. Their reasons for the disuse of the EMS system were analyzed and the findings show that 31% of the study subjects judged that it was inappropriate to use an ambulance, while 23.8% judged that it was faster to use a different transportation mode [5].

Rademaker et al. [23] classified the severity of ED patients into urgent, emergent, and routine. Routine severity refers to superficial injuries, simple lacerations, sprains, dislocations, chronic diseases, respiratory tract infections, gastrointestinal diseases, urinary system diseases, rashes, depression, and headache. Emergent severity was divided into A and B, wherein A referred to urgent and emergent cases which needed the use of ambulances, while B referred to routine and emergent cases which did not require the use of ambulances. In this research, the inappropriate use rate was 42% and the unmet use rate (equivalent to the underuse rate in this study) was 58%. Since the classification criteria differed between their research and this study, it is difficult to make a direct comparison. In addition, patients from different nations seem to have different characteristics since they have different insurance systems and either a paid or free EMS system.

According to the research in Australia, 2/3 of the elderly population aged 65 years and older used ambulances in nonurgent situations. Their results are similar to that of this study. These results may be attributable to the underlying diseases found in the elderly and their difficulty of movement [3, 24]. According to research by Kaufman et al. [4] in the US, 28,558 of 28,897 (98.8%) blunt trauma patients used ambulances. This is similar to the finding of this study where trauma patients had high rates of ambulance use. In cases of blunt trauma patients, the selected mode of transportation (such as whether to transport them by ambulance or a faster police car) differs depending on the situation.

According to research by Lee et al. [25], overestimation of the level of triage may occur since KTAS was based on the subjective evaluation of pain. In this study, using an ambulance is determined according to a patient's subjective judgment, and pain was already considered as a source of bias in the selection of the transportation mode for ED visits. Therefore, this did not influence the study results.

This study has some limitations: first, the primary outcome of this study was to find the overall frequency of use using already registered data. Therefore, only codified and collected data were analyzed, and it was impossible to take into account individual patients' special conditions, such as their clinical characteristics and underlying diseases. Regardless of severity, underlying diseases and personal situations can influence the use of the EMS system. Therefore, it is necessary for future research to analyze these as variables. Second, the research period only included data collected over three years. Given the changing trends of patients' EMS use, it is meaningful to conduct more long-term studies. Unfortunately, the available data system of ED patients only contains three years' worth of data. Therefore, it is necessary to analyze long-term trends when the data becomes available.

## 5. Conclusion

The adequacy of EMS system use and characteristics of ED patients were analyzed. The appropriate usage group accounted for 63.77% of the population, with 13.16% belonging to the group which used the EMS system, and 50.60% belonging to the group which did not use the EMS system. On the other hand, 36.23% of patients belonged to the inappropriate usage group 36.23%, with 28.13% belonging to the underuse group and 8.10% belonging to the overuse group. Higher rates of overuse were associated with male patients, old patients, cases of traffic accidents, those with accident-related insurance, trauma patients, and those who were brought to the ED at dawn. In contrast, lower rates of overuse were associated with female patients, cases of disease-induced ED visits, patients with an alert mental status, patients with KTAS level 3, those who were brought to the ED in the morning, and ED visits on weekends.

## Figures and Tables

**Table 1:** KTAS input rate by type of emergency medical centers.

	Type of emergency medical centers	Total	KTAS input *n* (%)
2016		9,127,979	5,558,617 (60.90)
	Regional centers	1,632,334	1,631,507 (99.95)
	Local centers	3,972,954	3,886,210 (97.82)
	Local institutions	3,522,691	40,900 (1.16)

2017		9,089,055	6,016,105 (66.19)
	Regional centers	1,800,371	1,800,347 (100.00)
	Local centers	3,791,033	3,757,485 (99.12)
	Local institutions	3,497,651	458,273 (13.10)

2018		9,266,269	6,763,970 (73.00)
	Regional centers	1,885,537	1,885,343 (99.99)
	Local centers	3,909,256	3,909,124 (100.00)
	Local institutions	3,471,476	969,503 (27.93)

Total		27,483,303	18,338,692 (66.73)

^*∗*^KTAS: Korean Triage and Acuity Scale.

**Table 2:** General characteristics of patients' comparison by ambulance use group, *n* (%).

	Appropriate EMS usage	Appropriate no EMS usage	Underuse	Overuse	*p*
	KTAS (1, 2, 3) and ambulance	KTAS (4, 5) and no ambulance	KTAS (1, 2, 3) and no ambulance	KTAS (4, 5) and ambulance	
	2,408,875 (13.16)	9,259,706 (50.6)	5,147,352 (28.13)	1,482,602 (8.10)	
Age mean ± S.D	58.20 ± 22.87	39.60 ± 26.89	34.15 ± 23.90	39.60 ± 26.89	*p* < 0.0001
Male (%)	53.81	49.83	51.28	49.83	*p* < 0.0001

*Disposition*					*p* < 0.0001
Discharge	1,047,286 (7.41%)	8,329,024 (58.95%)	3,638,366 (25.75%)	1,114,266 (7.89%)	
Transfer out	124,558 (43.77%)	46,651 (16.39%)	76,138 (26.75%)	37,240 (13.09%)	
Admission	1,143,853 (30.74%)	849,406 (22.83%)	1,407,848 (37.84%)	319,687 (8.59%)	
Death	86,919 (87.41%)	461 (0.46%)	11,132 (11.20%)	922 (0.93%)	
None	6,259 (9.66%)	34,164 (52.74%)	13,868 (21.41%)	10,487 (16.19%)	

*Insurance type*					*p* < 0.0001
Public	1,952,420 (12.12%)	8,334,443 (51.73%)	4,763,491 (29.57%)	1,061,109 (6.59%)	
Automobile	118,930 (16.53%)	335,009 (46.55%)	62,799 (8.73%)	202,931 (28.20%)	
Industrial injury	9,539 (21.81%)	22,486 (51.41%)	7,282 (16.65%)	4,433 (10.13%)	
Private	5 (10.42%)	22 (45.83%)	17 (35.42%)	4 (8.33%)	
Medical care type 1	231,391 (27.51%)	280,350 (33.33%)	192,303 (22.86%)	137,025 (16.29%)	
Medical care type 2	26,265 (15.44%)	82,748 (48.64%)	39,595 (23.27%)	21,515 (12.65%)	
Self-pay	56,839 (18.62%)	147,984 (48.49%)	56,179 (18.41%)	44,213 (14.49%)	
Others	11,047 (13.35%)	41,938 (50.68%)	20,062 (24.24%)	9,705 (11.73%)	
Unknown	2,439 (9.97%)	14,726 (60.21%)	5,624 (23.00%)	1,667 (6.82%)	

*Disease or injury*					*p* < 0.0001
Disease	1,882,159 (14.43%)	5,935,077 (45.51%)	4,536,214 (34.78%)	689,162 (5.28%)	
Injury	509,152 (10.12%)	3,183,726 (63.29%)	562,849 (11.19%)	774,762 (15.40%)	
Unknown	17,564 (7.79%)	140,903 (62.50%)	48,289 (21.42%)	18,678 (8.29%)	

*Route of arrival*					*p* < 0.0001
Direct	1,853,679 (11.41%)	8,696,332 (53.53%)	4,335,211 (26.69%)	1,360,492 (8.37%)	
Transfer in	552,549 (31.92%)	431,373 (24.92%)	626,772 (36.20%)	120,542 (6.96%)	
Referral from outpatient	1,618 (0.54%)	118,864 (39.65%)	178,317 (59.48%)	970 (0.32%)	
Others	825 (18.72%)	1,709 (38.77%)	1,422 (32.26%)	452 (10.25%)	
Unknown	204 (1.17%)	11,428 (65.65%)	5,630 (32.34%)	146 (0.84%)	

*Mode of transport*					*p* < 0.0001
Public EMS	1,828,405 (57.80%)	0	0	1,334,809 (42.2%)	
Other hospital ambulances	142,140 (77.28%)	0	0	41,789 (22.72%)	
Private ambulance service	438,330 (80.53%)	0	0	106,004 (19.47%)	
Public transportation (e.g., police car)	0	11,943 (62.83%)	7,066 (37.17%)	0	
Aeromedical transport	0	4,834 (36.56%)	8,387 (63.44%)	0	
Other cars	0	9,112,394 (64.31%)	5,056,599 (35.69%)	0	
Walk-in	0	106,108 (65.81%)	55,117 (34.19%)	0	
Others	0	12,534 (47.32%)	13,956 (52.68%)	0	
Unknown	0	11,893 (65.63%)	6,227 (34.37%)	0	

*Type of emergency medical centers*					*p* < 0.0001
Regional centers	890,035 (16.74%)	2,275,717 (42.81%)	1,787,642 (33.63%)	362,725 (6.82%)	
Local centers	1,426,363 (12.35%)	6,050,092 (52.40%)	3,074,910 (26.63%)	994,295 (8.61%)	
Local institutions	92,477 (6.44%)	933,897 (65.00%)	284,800 (19.82%)	125,582 (8.74%)	

*Initial triage level*					*p* < 0.0001
1	166,114 (84.69%)	0	30,030 (15.31%)	0	
2	549,418 (51.93%)	0	508,539 (48.07%)	0	
3	1,693,343 (26.87%)	0	4,608,783 (73.13%)	0	
4	0	7,347,225 (85.02%)	0	1,294,064 (14.98%)	
5	0	1,912,481 (91.03%)	0	188,538 (8.97%)	

^*∗*^KTAS: Korean Triage and Acuity Scale.

**Table 3:** Arrival time and month of patients, *n* (%).

	Appropriate EMS usage	Appropriate no EMS usage	Underuse	Overuse	*p*
	KTAS (1, 2, 3) and ambulance	KTAS (4, 5) and no ambulance	KTAS (1, 2, 3) and no ambulance	KTAS (4, 5) and ambulance	
	2,408,875 (13.16)	9,259,706 (50.6)	5,147,352 (28.13)	1,482,602 (8.10)	
*Arrival time*					*p* < 0.0001
00	80,374 (11.20)	381,268 (26.35)	189,027 (53.15)	66,700 (9.30)	
01	68,186 (12.07)	286,967 (26.93)	152,173 (50.78)	57,808 (10.23)	
02	59,473 (12.70)	231,271 (27.40)	128,258 (49.40)	49,127 (10.49)	
03	53,299 (13.26)	194,297 (27.98)	112,476 (48.34)	41,878 (10.42)	
04	50,033 (14.07)	166,685 (29.00)	103,089 (46.89)	35,686 (10.04)	
05	51,173 (15.16)	153,390 (29.57)	99,840 (45.43)	33,203 (9.83)	
06	59,713 (16.05)	165,505 (30.34)	112,889 (44.48)	33,967 (9.13)	
07	75,215 (16.82)	198,421 (29.86)	133,503 (44.38)	39,959 (8.94)	
08	96,110 (17.73)	239,457 (28.59)	154,965 (44.18)	51,478 (9.50)	
09	122,525 (16.48)	334,872 (29.48)	219,206 (45.13)	67,009 (9.01)	
10	139,688 (15.90)	395,380 (30.78)	270,499 (44.99)	73,203 (8.33)	
11	140,992 (15.92)	396,309 (31.34)	277,505 (44.76)	70,549 (7.97)	
12	133,956 (16.43)	361,569 (31.07)	253,287 (44.37)	66,239 (8.13)	
13	128,867 (16.05)	372,004 (29.17)	234,244 (46.32)	67,917 (8.46)	
14	127,844 (15.20)	397,808 (29.08)	244,551 (47.31)	70,684 (8.41)	
15	126,603 (14.91)	405,753 (28.98)	246,103 (47.77)	70,849 (8.34)	
16	123,608 (14.16)	424,905 (29.04)	253,483 (48.68)	70,825 (8.11)	
17	119,946 (12.94)	475,518 (28.29)	262,252 (51.29)	69,363 (7.48)	
18	119,786 (12.04)	536,107 (26.74)	266,164 (53.87)	73,192 (7.35)	
19	120,495 (10.45)	652,310 (26.26)	302,800 (56.58)	77,368 (6.71)	
20	112,768 (9.38)	700,156 (26.24)	315,511 (58.23)	74,022 (6.16)	
21	106,409 (9.07)	685,711 (26.14)	306,518 (58.47)	74,047 (6.31)	
22	100,550 (9.46)	611,312 (25.92)	275,415 (57.53)	75,309 (7.09)	
23	91,262 (10.26)	492,641 (26.25)	233,594 (55.37)	72,220 (8.12)	
*Arrival day*					*p* < 0.0001
Monday	372,094 (14.55)	1,214,418 (29.59)	756,618 (47.49)	213,997 (8.37)	
Tuesday	342,115 (14.76)	1,098,608 (29.05)	673,070 (47.41)	203,334 (8.78)	
Wednesday	339,208 (15.01)	1,062,558 (29.02)	655,829 (47.02)	201,974 (8.94)	
Thursday	341,922 (15.29)	1,041,140 (29.06)	649,958 (46.54)	203,832 (9.11)	
Friday	349,901 (15.22)	1,075,839 (28.72)	660,187 (46.81)	212,625 (9.25)	
Saturday	337,521 (11.48)	1,589,544 (26.72)	785,462 (54.08)	226,931 (7.72)	
Sunday	326,114 (8.84)	2,177,599 (26.19)	966,228 (59.02)	219,909 (5.96)	
*Arrival month*					*p* < 0.0001
1	194,566 (13.41)	727,086 (28.86)	418,615 (50.12)	110,287 (7.60)	
2	173,628 (12.85)	697,128 (27.95)	377,610 (51.59)	102,832 (7.61)	
3	186,845 (13.90)	660,176 (28.67)	385,433 (49.11)	111,956 (8.33)	
4	193,199 (13.55)	705,037 (28.81)	410,672 (49.46)	116,445 (8.17)	
5	206,075 (12.98)	800,890 (28.58)	453,851 (50.44)	126,915 (7.99)	
6	201,327 (13.23)	759,983 (28.64)	435,736 (49.94)	124,598 (8.19)	
7	212,450 (13.19)	804,866 (28.62)	460,862 (49.98)	132,189 (8.21)	
8	211,854 (13.27)	797,896 (28.24)	450,272 (50.04)	134,653 (8.45)	
9	202,369 (12.61)	835,471 (27.13)	435,571 (52.04)	131,985 (8.22)	
10	204,729 (13.30)	792,572 (26.76)	411,786 (51.50)	129,765 (8.43)	
11	199,155 (14.27)	679,511 (28.35)	395,723 (48.69)	121,310 (8.69)	
12	222,948 (11.90)	999,090 (27.30)	511,221 (53.34)	139,667 (7.46)	

^*∗*^KTAS: Korean Triage and Acuity Scale.

## Data Availability

The data used to support the findings of this study were supplied by NEDIS, Korea, under license and so cannot be made freely available. Requests for access to these data should be made to NEDIS, Korea.
